# Genomics and Genetics of Aging

**DOI:** 10.2174/138920212803251409

**Published:** 2012-11

**Authors:** Matt Kaeberlein

The biology of aging is immensely complex. Aging occurs at the molecular, cellular, tissue, organ, and organismal level and drives the onset and progression of nearly every medically relevant disease in developed nations. Aging is influenced by multiple genetic and environmental factors, some of which are conserved across broad evolutionary distance.

This special issue is aimed at presenting the complexity of aging in a manner accessible to a broad readership, with particular emphasis on genomic and systems biology approaches to understand the aging process. Such approaches are needed to better model the pathways and interactions that underlie the aging process.

The issue begins with an overview of large-scale and genome-wide studies of aging by McCormick and Kennedy. A synthesis of studies from multiple model systems including *Saccharomyces cerevisiae*, *Caenorhabditis elegans*, *Drosophila melanogaster*, and *Mus musculus *is presented.

Yanos *et al.* focus in detail on what has been gleaned from genomic RNAi longevity screens in *C. elegans*. Hundreds of genes that limit lifespan have been identified from such studies, which has provided a wealth of information but also the challenge of trying to understand how these genetic factors interact to influence aging. The importance of the specific longevity pathways involved, including insulin-like signaling, dietary restriction, mRNA translation, and mitochondrial function is discussed.

Mitochondria have long been thought to contribute to aging through the production of damaging reactive oxygen species; however, as Hwang *et al. *describe, more recent findings have forced a reevaluation of this model. It is now clear that certain forms of mitochondrial stress are compatible with longer and healthier life in organisms from yeast to mice. Specific models accounting for these observations, including possible involvement of the hypoxic response transcription factor, HIF-1, are described in detail.

Although chromatin modifying factors, such as histone deacetylases, were among the first enzymes to be shown to modulate lifespan in model organisms, the full importance of epigenetics in aging has only recently been appreciated. Liu and Zhou present an overview of the importance of chromatin remodeling in aging and the response to DNA damage.

MicroRNAs represent yet another level of complexity in the aging process. These small RNAs are able to regulate the expression of numerous mRNAs, some of which directly influence aging and age-related diseases. Jung and Suh describe what we know about the importance of microRNAs in aging and how this exciting new field is just starting to become explored.

The last review in this special issue by Hou *et al.* brings things together nicely with a systems biology perspective of aging. In order to model the immense complexity of aging, we require systems-level approaches. This review describes how several groups are trying to tackle this problem and some of the new insights that have already been gained.

I would like to thank all of the authors and reviewers for their efforts in putting together such a timely and informative special issue.

